# Effects of Different Types of Pathogenic Variants on Phenotypes of Familial Hypercholesterolemia

**DOI:** 10.3389/fgene.2022.872056

**Published:** 2022-04-11

**Authors:** Hayato Tada, Nobuko Kojima, Kan Yamagami, Akihiro Nomura, Atsushi Nohara, Soichiro Usui, Kenji Sakata, Noboru Fujino, Masayuki Takamura, Masa-Aki Kawashiri

**Affiliations:** ^1^ Department of Cardiovascular Medicine, Graduate School of Medical Sciences, Kanazawa University, Kanazawa, Japan; ^2^ Department of Clinical Genetics, Ishikawa Prefectural Central Hospital, Kanazawa, Japan

**Keywords:** familial hypercholesterolemia, LDL cholesterol, genetics, LDL receptor (LDLR), protein-truncating variants

## Abstract

**Objective:** It has been shown that pathogenic variants are associated with poor clinical outcomes in patients with familial hypercholesterolemia (FH). However, data on the effect of different types of pathogenic variants on FH phenotype is limited.

**Methods:** We retrospectively investigated the associations between genotypes and phenotypes, including low-density lipoprotein (LDL) cholesterol level and the occurrence of major adverse cardiac events (MACEs), defined as cardiovascular death, myocardial infarction, unstable angina, or coronary artery revascularization, in patients with FH (N = 1,050, male/female = 490/560). Based on genotype, the patients were divided into the following three groups: patients without pathogenic variants, patients with missense variants, and patients with protein-truncating variants (PTVs). Cox proportional hazard model was used to identify the factors associated with MACEs.

**Results:** The median follow-up duration was 12.6 years (interquartile range = 9.5–17.9 years). There were 665 patients with FH-mutation (277 patients with missense variants and 388 patients with PTVs) and 385 patients without FH-mutation. Over the follow-up duration, 175 MACEs were observed. We identified 89 different pathogenic variants in the 665 patients with FH. LDL cholesterol level was found to be significantly higher in patients with PTVs (256 mg/dl) than in patients with missense variants (236 mg/dl) and patients without pathogenic variants (216 mg/dl). It was also found that PTVs and missense variants are significantly associated with MACEs (hazard ratio [HR] = 1.58, 95% confidence interval [CI] = 1.08–2.08, *p* = 0.0033 and HR = 3.24, 95% CI = 2.12–4.40, *p* = 3.9 × 10^−6^, respectively), independent of classical risk factors.

**Conclusion:** Pathogenic variants, especially PTVs, are significantly associated with poor outcomes in patients with FH. Genetic testing is useful for the diagnosis and risk stratification of patients with FH.

## Highlights

• Pathogenic variants are associated with higher risk for CVD among patients with FH.
• LDL cholesterol level differs according to types of pathogenic variants among patients with FH.
• Prognosis differs according to types of pathogenic variants among patients with FH.

## 1 Introduction

Familial hypercholesterolemia (FH) is one of the most common Mendelian disorders, and its incidence in the general population is reported to be one in 300 (Beheshti et al., 2020; Hu et al., 2020). FH is caused by genetic variants associated with low-density lipoprotein (LDL) metabolism, such as the LDL receptor (*LDLR*), apolipoprotein B (*APOB*), proprotein convertase subtilisin/kexin type 9 (*PCSK9*), and *LDLR* adaptor protein 1 (*LDLRAP1*) (Mabuchi, 2017). Typically, FH is diagnosed based on clinical criteria (Austin et al., 2004; Harada-Shiba et al., 2018; Tada et al., 2021a); however, it was reported that pathogenic variants may be associated with increased risk of coronary artery disease (Khera et al., 2016; Tada et al., 2017). Therefore, genetic testing is recommended for the diagnosis and risk stratification of patients with FH. More than 2000 pathogenic variants have been identified worldwide, and most of them are *LDLR* variants (Nohara et al., 2021). Based on genotype, pathogenic variants can be classified as missense variants (which can estimate residual LDLR activity) or protein-truncating variants (PTVs), which may have lost their LDLR function. Only a few studies have investigated the effects of pathogenic variants on the phenotype of patients with FH (Tada et al., 2020). The aim of this study was to investigate the associations between genotypes and phenotypes, including LDL cholesterol level and occurrence of major adverse cardiac events (MACEs) in patients with FH diagnosed using clinical diagnostic criteria.

## 2 Materials and Methods

### 2.1 Study Population

We evaluated the data of 2011 patients with FH diagnosed clinically using the Japan Atherosclerosis Society (JAS) 2017 criteria at Kanazawa University Hospital between 1990 and 2020. All the patients in this study fulfilled at least two of the three essential clinical criteria stipulated by the JAS for FH diagnosis. The criteria are as follows: 1) LDL cholesterol level ≥180 mg/dl, 2) tendon xanthoma on the backs of the hands, elbows, knees, or other areas; Achilles tendon hypertrophy or Achilles tendon thickness on X-ray ≥ 9 mm; or xanthoma tuberosum, and 3) family history of FH or premature coronary artery disease diagnosed in a first- or second-degree relative. Nine hundred and sixty-one patients were excluded due to missing data (such as data on blood lipids and genetic analysis or data on homozygous and compound heterozygous FH. Finally, 1,050 patients were included in this study (Supplementaary Figure S1).

### 2.2 Clinical Data Assessment

We defined hypertension as systolic blood pressure ≥140 mmHg, diastolic blood pressure ≥90 mmHg, or use of antihypertensive agents. Further, we used the definition of diabetes given by the Japan Diabetes Society (Araki et al., 2020). Smoking status was defined as a current smoking status. Cardiovascular disease (CVD) was defined as angina pectoris, myocardial infarction, or severe stenotic region(s) in the coronary artery (≥75% stenosis), identified on angiography or computed tomography. Serum levels of total cholesterol, triglycerides, and high-density lipoprotein cholesterol were determined enzymatically using automated instrumentation. LDL cholesterol level was calculated using the Friedewald formula if triglyceride level was <400 mg/dl; otherwise, it was determined enzymatically.

### 2.3 Genetic Analysis

We assessed genotypes using a next-generation sequencer. In brief, the coding regions of *LDLR*, *APOB, PCSK9*, and *LDLRAP1* were sequenced as described in a previous study (Tada et al., 2018). Further, copy number variations at the *LDLR* were assessed using the eXome Hidden Markov Model software as described in an earlier study (Yamamoto et al., 2016). We evaluated the pathogenicity of the genetic variants according to the standard American College of Medical Genetics and Genomics criteria (Richards et al., 2015). We classified pathogenic variants as missense variants or PTVs, which include frameshift variants, large deletion or duplication variants, nonsense variants, and splice site variants.

### 2.4 Ethical Considerations

This study was approved by the Ethics Committee of Kanazawa University. All procedures were conducted in accordance with the ethical standards of the Human Research Committee (institutional and national) and the Helsinki Declaration (1975, revised in 2008). Informed consent for genetic analysis was obtained from all the study participants.

### 2.5 Statistical Analysis

Categorical variables were reported as numbers and percentages, and they were compared using Fisher’s exact test or chi-square test. Normally distributed continuous variables were reported as means ± standard deviations. Not normally distributed continuous variables were reported as medians and interquartile ranges. Mean values of continuous variables were compared using Student’s t-test for independent variables, and median values were compared using non-parametric Wilcoxon–Mann–Whitney rank sum test. Chi-square or Fisher’s post-hoc test was used for categorical variables as indicated. Cox proportional hazard model was used to assess relationships between all the variables. Cumulative Kaplan–Meier survival curves starting at baseline were constructed to compare times to the first MACE. All statistical analyses were conducted using R statistics (https://www.r-project.org). *p*-values < 0.05 were considered statistically significant.

## 3 Results

### 3.1 Clinical Characteristics

The clinical characteristics of the study participants are shown in Table 1. The mean age of patients was 49 years, and almost half of the patients were men. The median LDL cholesterol level at baseline was 244 mg/dl, and it decreased to 110 mg/dl at follow-up. A total of 776 patients (73.9%) had a family history of FH and/or premature CVD. Furthermore, 290 patients (27.6%) had a history of CVD. Upon division of patients into two groups based on the occurrence of MACEs, we observed significant differences in all variables (except family history of FH and/or premature CVD) between the two groups. In addition, we observed differences in the trend of characteristics; for example, we observed, following division of patients with pathogenic mutations into three groups based on genotype, that the proportion of patients with diabetes decreased (Supplementary Table S1). The medical treatments administered during follow-up are summarized in Table 2. Statin therapy, frequently followed by ezetimibe and colestimide therapy, was administered to most of the patients.

**TABLE 1:** Baseline characteristics.

Variable	All	MACE	NO-MACE	*p*-value
	(N = 1,050)	(N = 175)	(N = 875)	
Age (years)	49 ± 16	59 ± 15	46 ± 17	<2.2 × 10^−16^
Male gender (%)	490 (46.7%)	115 (65.7%)	375 (42.9%)	5.0 × 10^−8^
Hypertension (%)	250 (23.8%)	114 (65.1%)	136 (15.5%)	<2.2 × 10^−16^
Diabetes (%)	83 (7.9%)	45 (25.7%)	38 (4.3%)	0.0016
Smoking (%)	224 (21.3%)	96 (54.9%)	128 (14.6%)	<2.2 × 10^−16^
Total cholesterol level (mg/dl)	326 [268–365]	340 [280–382]	322 [261–358]	0.00021
Triglyceride level (mg/dl)	113 [76–177]	144 [91–185]	126 [80–171]	0.0045
HDL cholesterol level (mg/dl)	47 [43–51]	45 [41–49]	48 [44–52]	0.0019
LDL cholesterol level (at baseline, mg/dL)	244 [208–279]	256 [218–294]	240 [204–270]	0.0011
LDL cholesterol level (at follow-up, mg/dL)	110 [96–120]	102 [90–116]	112 [96–121]	0.0024
Family history of FH and/or premature CVD (%)	776 (79.9%)	140 (80.0%)	636 (72.7%)	0.055
Pathogenic variants of FH (%)	777 (74.0%)	153 (87.4%)	624 (71.3%)	1.4 × 10^−5^
History of CVD (%)	290 (27.6%)	141 (80.6%)	149 (17.0%)	<2.2 × 10^−16^

MACE, major adverse cardiac event; FH, familial hypercholesterolemia; CVD, cardiovascular disease; HDL, high-density lipoprotein; LDL, low-density lipoprotein.

**TABLE 2:** Medical therapies.

Lipid-lowering therapy	All	Without pathogenic variants	With missense variants	With PTVs
	(N = 1,050)	(N = 385)	(N = 277)	(N = 388)
Statins (%)	1,025 (97.6%)	380 (98.7%)	265 (95.7%)	380 (97.9%)
Ezetimibe (%)	644 (61.3%)	198 (51.4%)	160 (57.8%)	286 (73.7%)
Colestimide (%)	243 (23.1%)	30 (7.8%)	70 (25.3%)	143 (36.9%)
Probucol (%)	2 (0.2%)	0 (0.0%)	1 (0.4%)	1 (0.3%)
PCSK9 inhibitor (%)	45 (4.3%)	3 (0.8%)	13 (4.7%)	29 (7.5%)
LDL apheresis (%)	2 (0.2%)	0 (0.0%)	0 (0.0%)	2 (0.5%)
Fibrates (%)	6 (0.6%)	3 (0.8%)	2 (0.7%)	1 (0.3%)
n-3 PUFAs (%)	10 (1.0%)	5 (1.3%)	1 (0.4%)	4 (1.0%)

PTV, protein-truncating variants; PCSK9, proprotein convertase subtilisin/kexin type 9; PUFA, polyunsaturated fatty acid; LDL, low-density lipoprotein.

### 3.2 Mutation Distributions

**FIGURE 1:**
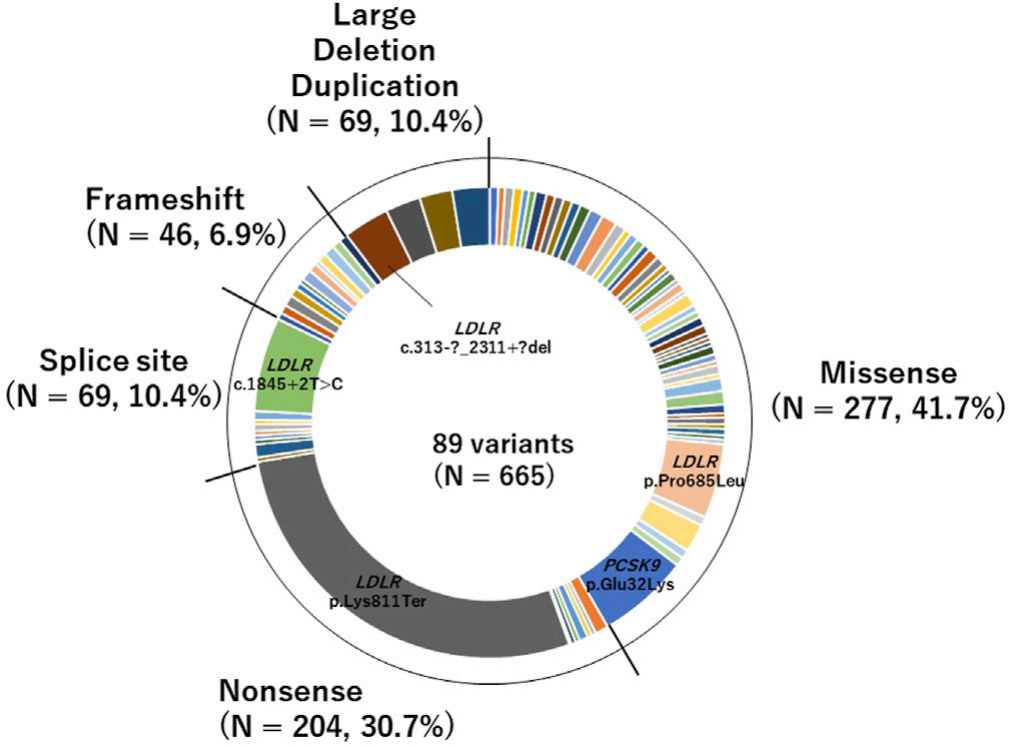
Pie chart of pathogenic variants identified in this study of the pathogenic variants, 277 were classified as missense variants, and 388 were classified as PTVs (46 were frameshift variants, 69 were large deletion or duplication variants, 204 were nonsense variants, and 69 were splice site variants).

We identified 89 pathogenic variants in 665 patients. Of the pathogenic variants, 277 were classified as missense variants, and 388 were classified as PTVs (46 were frameshift variants, 69 were large deletion or duplication variants, 204 were nonsense variants, and 69 were splice site variants; Figure 1). The details are shown in Supplementary Table S2.

### 3.3 LDL Cholesterol Level According to Genotype

**FIGURE 2:**
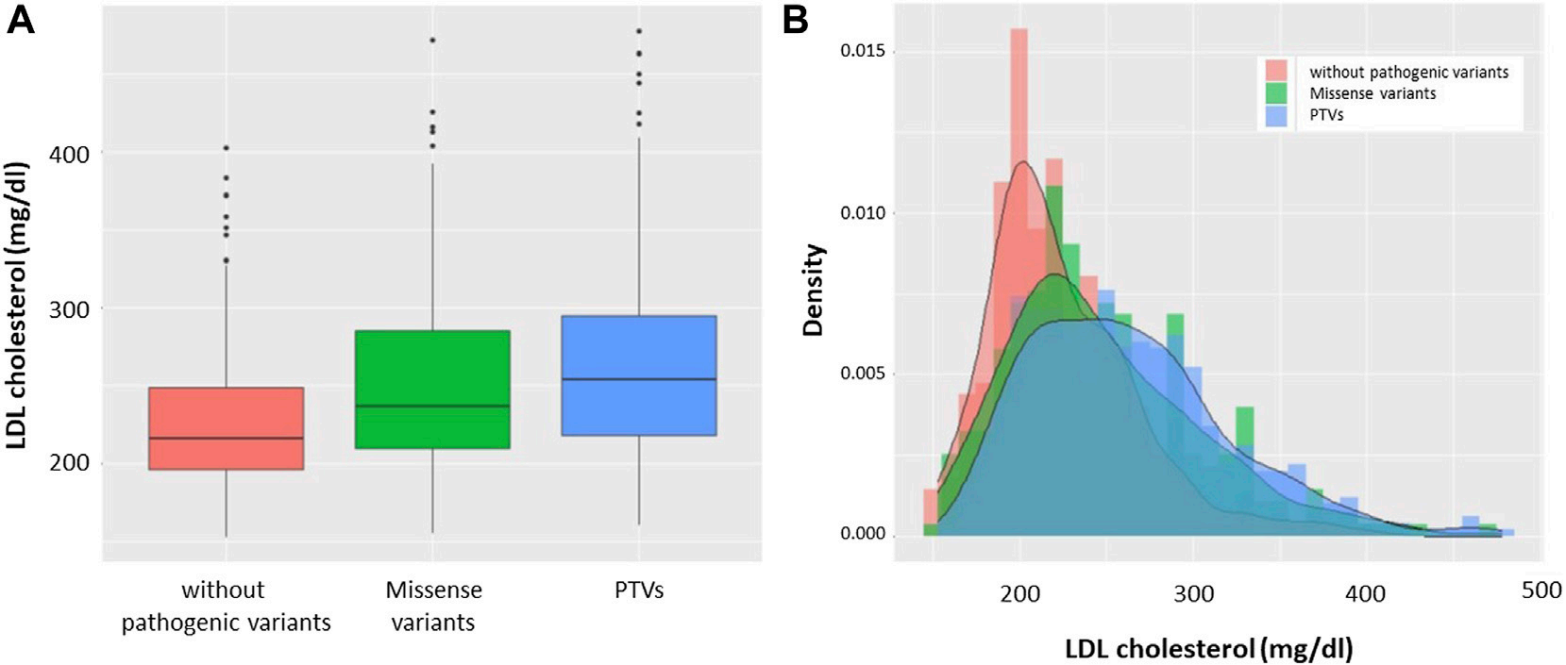
LDL cholesterol levels according to mutation status. **(A)** Boxplots: Red indicates patients without pathogenic variants. Green indicates patients with missense variants. Blue indicates patients with PTVs. **(B)** Histograms with density: Red indicates patients without pathogenic variants. Green indicates patients with missense variants. Blue indicates patients with PTVs.

Based on genotype, we divided the patients into the following three groups: patients without pathogenic variants, patients with missense variants, and patients with PTVs. We found that LDL cholesterol level was highest in patients with PTVs, and patients with missense variants were found to have higher LDL cholesterol levels than patients without pathogenic variants (Figure 2A). The median LDL cholesterol levels of patients without pathogenic variants, patients with missense variants, and patients with PTVs were 216 mg/dl, 236 mg/dl, and 256 mg/dl, respectively. The LDL cholesterol levels of the three groups showed deformed trimodal distributions (Figure 2B).

### 3.4 Factors Associated With MACEs

Over the median follow-up duration of 12.6 years, 175 patients had MACEs, which include CVD-associated death, myocardial infarction, unstable angina, and staged revascularization (Table 3). Using Cox proportional hazard model, we assessed factors associated with MACEs and found that age (hazard ratio [HR] = 1.09, 95% confidence interval [CI] = 1.04–1.14, *p* = 2.2 × 10^−14^), male gender (HR = 1.58, 95% CI = 1.08–2.08, *p* = 0.0079), hypertension (HR = 3.11, 95% CI = 2.10–4.25, *p* = 6.9 × 10^−6^), diabetes (HR = 2.44, 95% CI = 1.46–3.52, *p* = 0.0021), smoking (HR = 2.56, 95% CI = 1.56–3.18, *p* = 0.00041), LDL cholesterol level (per 10 mg/dl; HR = 1.01, 95% CI = 1.00–1.02, *p* = 0.022), and prior CVD (HR = 3.45, 95% CI = 2.02–4.80, *p* < 2.2 × 10^−16^) are significantly associated with MACEs (Table 4). We also found that missense variants and PTVs are associated with MACEs (HR = 1.58, 95% CI = 1.08–2.08, *p* = 0.0033 and HR = 3.24, 95% CI = 2.12–4.40, *p* = 3.9 × 10^−6^, respectively).

**TABLE 3:** Types of MACEs.

Type of MACE	All (N = 1,050)
CVD-associated death	56 (5.3%)
Myocardial infarction	24 (2.3%)
Unstable angina	33 (3.1%)
Staged revascularization	62 (5.9%)
Total	175 (16.7%)

MACE, major adverse cardiac event; CVD, cardiovascular disease.

**TABLE 4:** Factors associated with MACEs.

Variable	HR	95% CI	*p*-value
Age (per year)	1.09	1.04–1.14	2.2 × 10^−14^
Male gender (yes versus no)	1.58	1.08–2.08	0.0079
Hypertension (yes versus no)	3.11	2.10–4.25	6.9 × 10^−6^
Diabetes (yes versus no)	2.44	1.46–3.52	0.0021
Smoking (yes versus no)	2.56	1.56–3.18	0.00041
LDL cholesterol level (per 10 mg/dl)	1.01	1.00–1.02	0.022
Prior CVD (yes versus no)	3.45	2.02–4.80	<2.2 × 10^−16^
Missense variants (versus no pathogenic variants)	1.58	1.08–2.08	0.0033
PTVs (versus no pathogenic variants)	3.24	2.12–4.40	3.9 × 10^−6^

HR, hazard ratio; CI, confidence interval; CVD, cardiovascular disease; PTV, protein-truncating variants; LDL, low-density lipoprotein.

### 3.5 Prognosis According to Genotype

**FIGURE 3:**
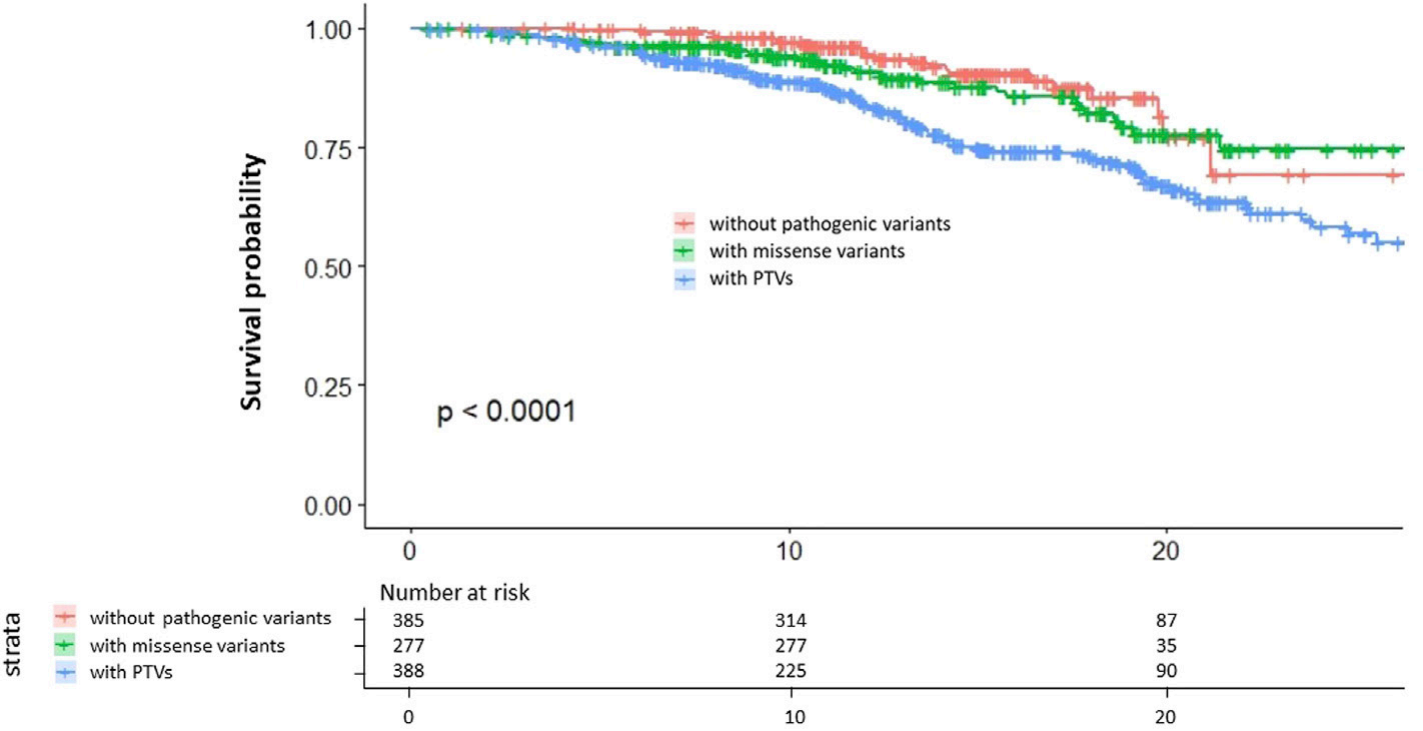
Kaplan–Meier survival curves. Red indicates patients without pathogenic variants. Green indicates patients with missense variants. Blue indicates patients with PTVs.

We assessed the survival curve and found that patients with PTVs have the worst outcome of the three groups and that patients with missense variants have worse outcomes than patients without pathogenic variants (Figure 3).

## 4 Discussion

In this study, we evaluated the effects of pathogenic variants on the clinical phenotypes of FH and found that 1) LDL cholesterol level is highest in patients with PTVs and higher in patients with missense variants than in patients without pathogenic variants, 2) prognosis is worst in patients with PTVs and worse in patients with missense variants than in patients without pathogenic variants.

There are many factors, including traditional risk factors and genetic factors that have been associated with CVD in patients with FH. Indeed, a clinical risk score named Montreal-FH-score comprising traditional risk factors has been one of the useful tools predicting CVD risk in patients with FH (Paquette et al., 2017). In addition, we and others have shown that rare and common genetic variations contributed to increase or decrease risks for CVD among patients with FH on top of FH-mutation (Tada et al., 2018; Fahed et al., 2020). Based on different standpoints, including diagnosis, cascade screening, and risk stratification, genetic testing is currently recommended for FH (Sturm et al., 2018). Patients with FH are associated with significantly high risk of CVD due to cumulative exposure to elevated LDL cholesterol level (Tada et al., 2021b). Thus, early diagnosis and intervention are considered vital. Previous studies reveal that FH diagnosis is improved by early treatment of patients (Luirink et al., 2019; Tada et al., 2021c). Since patients with FH typically do not have tendon xanthomas (one of the most important clinical diagnostic criteria) in childhood, genetic testing is essential for early diagnosis of FH (Tada et al., 2021d; Matsunaga et al., 2021; Nagahara et al., 2021). Furthermore, given the criteria of pathogenicity of genetic variants and the catalog of pathogenic variants of FH, there is a growing demand for further risk stratification of patients with FH based on genotype (Chora et al., 2022). The growing demand is natural in this personalized medicine era, when phenotypes can be estimated using genotypes and the best treatments for many inherited diseases can be determined based on genotype (Sukrithan et al., 2019; Pujol et al., 2021; Tada, 2021). We expect that patients with PTVs will receive intensive treatment and that cascade screening will be recommended for patients with pathogenic variants, especially PTVs, to identify patients with high CVD risk.

This study has several limitations. First, this is a retrospective study conducted in a single center. Therefore, the study findings may not be applicable to other patients. However, our institute has a long history of treating patients with FH and has one of the largest databases in Japan. Second, we could not account for treatments administered during follow-up, and this may affect the study results. Third, many patients were excluded from analysis due to missing data or loss to follow-up. This exclusion may also affect study results. When we compared the baseline characteristics (only the key variables, including age, gender, and LDL cholesterol) between study subjects and those excluded. We found that the mean age of the patients excluded from this study was significantly younger than that of patients included in this study, although there were no significant differences between these 2 groups in gender and LDL cholesterol (Supplementary Table S2). Fourth, in this study, functional analysis was not performed to validate the pathogenicity of genetic variants. Fifth, polygenic factors were not considered in this study. Sixth, we did not account for functional analyses of the variants, especially in missense variants. In fact, some of the missense variants, such as p.Ser177Leu, p.Glu228Gln, p.Asp266Asn, and p.Val429Leu have been considered as “null alleles” based on functional assays (Hobbs et al., 1992). However, the number of individuals with these variants were so small that it is unlikely to affect our results. In addition, it is difficult to classify all of the variants of this study clearly because of lack of such data. Furthermore, a simple classification of PTVs appears to have great impact on phenotypes in our data. Accordingly, we believe that it is beneficial for us to divide PTVs and missense variants in case of FH. Seventh, we did not fully observe other atherosclerotic disease, such as cerebrovascular disease, peripheral artery disease and aortic valve stenosis unless they exhibited any symptoms suggesting these conditions. Further studies will full assessments of these conditions will be useful to estimate their comprehensive risk assessments.

In conclusion, pathogenic variants, especially PTVs, are significantly associated with poor outcomes in patients with FH. Genetic testing is useful for the diagnosis and further risk stratification of patients with FH.

## Data Availability

The datasets presented in this article are not readily available because the IRB of Kanazawa University did not give us an approval to deposit genomic data even in a public repository. Requests to access the datasets should be directed to the corresponding author, HT.
