# Supplementary material for: Effects of Different Types of Pathogenic Variants on Phenotypes of Familial Hypercholesterolemia
Source: Front Genet. 2022 Apr 11;13:872056. doi: 10.3389/fgene.2022.872056 (PMC9035489; doi:10.3389/fgene.2022.872056)
Supplement: Supplementary file 1 [file Presentation1.pdf]

Supplementary Material

Supplemental Figure 1. STROBE flow diagram of patient selection

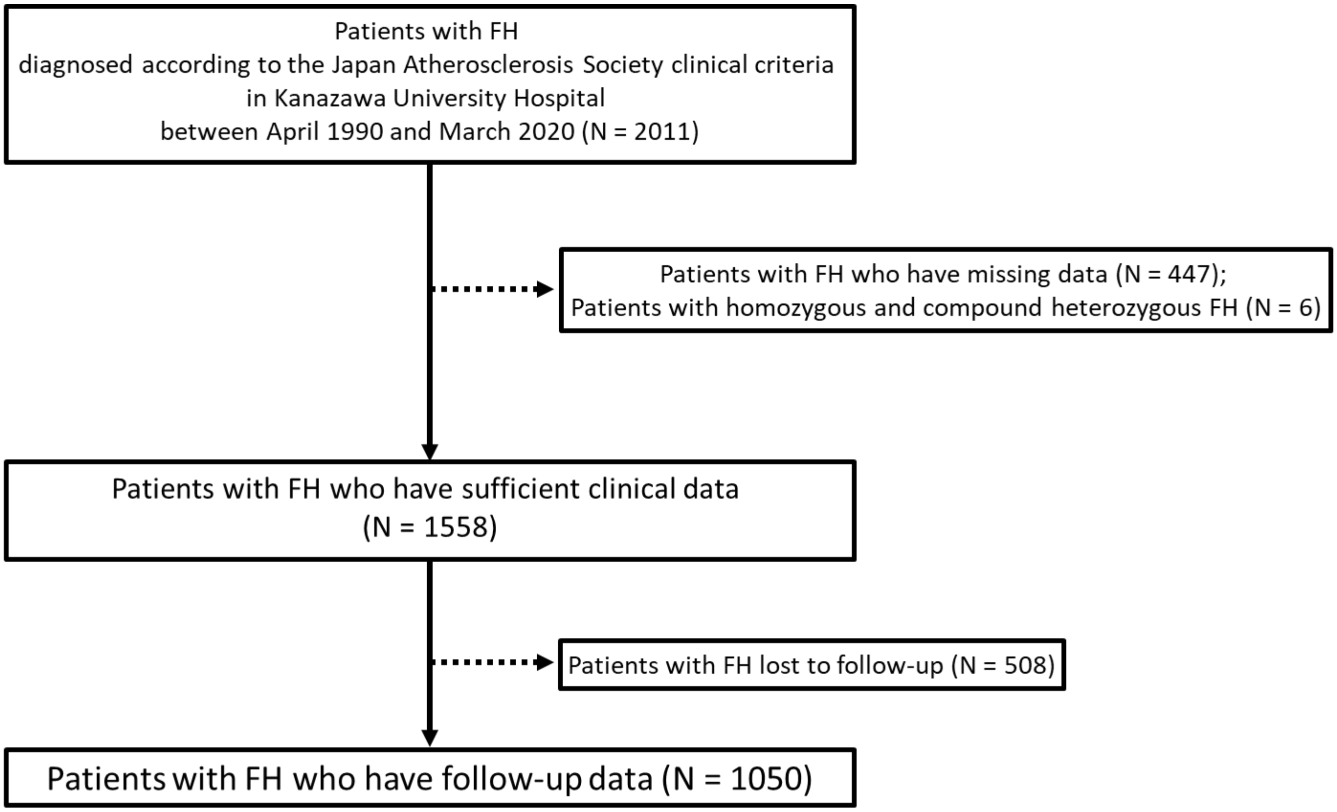

The flow diagram shows the process of patient selection.

Supplemental Table 1. Baseline characteristics according to genotype

	All	without pathogenic variants	with missense variants	with PTVs
	(N = 1050)	(N = 385)	(N = 277)	(N = 388)
Age (years)	49 ± 16	52 ± 16	48 ± 17	46 ± 17
Male gender (%)	490 (46.7%)	200 (51.9%)	127 (45.8%)	163 (42.0%)
Hypertension (%)	250 (23.8%)	99 (25.7%)	66 (23.8%)	85 (21.9%)
Diabetes (%)	83 (7.9%)	53 (13.8%)	14 (5.1%)	16 (4.1%)
Smoking (%)	224 (21.3%)	90 (23.4%)	56 (20.2%)	78 (20.1%)
Total cholesterol level (mg/dL)	326 [268–365]	318 [244–344]	328 [279–377]	348 [290–392]
Triglyceride level (mg/dL)	113 [76–177]	142 [90–190]	125 [80–175]	104 [78–170]
HDL cholesterol level (mg/dL)	47 [43–51]	49 [44–52]	48 [43–50]	45 [41–49]
LDL cholesterol level (at baseline, mg/dL)	244 [208–279]	216 [196–248]	236 [219–285]	256 [218–295]
LDL cholesterol level (at follow-up, mg/dL)	110 [96–120]	112 [98–1120]	114 [94–122]	106 [90–118]
Family history of FH and/or premature CVD (%)	776 (73.9%)	277 (71.9%)	193 (69.7%)	306 (78.9%)
History of CVD (%)	290 (27.6%)	97 (25.2%)	69 (24.9%)	124 (32.0%)

PTV: protein-truncating variant, CVD: cardiovascular disease, HDL: high-density lipoprotein, LDH: low-density lipoprotein, FH: familial hypercholesterolemia

Supplemental Table 2. Mutations identified in FH genes

Gene	Nucleotide change	Mutation type	Effect on protein	Number of patients	ACMG
LDLR	c.14G>A	Missense	p.Gly5Asp	4	PM1/PM2/PP3/PP4 Likely pathogenic
LDLR	c.68-1G>C	Splice site	NA	2	PVS1/PM2/PM4/PP1/PP5 Pathogenic
LDLR	c.130T>G	Missense	p.Trp44Gly	3	PM1/PM2/PP3/PP4 Likely pathogenic
LDLR	c.137G>A	Missense	p.Cys46Tyr	4	PM1/PM2/PP3/PP5 Likely pathogenic
LDLR	c.191-2A>G	Splice site	NA	6	PVS1/PM2/PM4/PP1 Pathogenic
LDLR	c.283T>G	Missense	p.Cys95Gly	4	PM1/PM2/PP3/PP4 Likely pathogenic
LDLR	c.284G>T	Missense	p.Cys95Phe	3	PM2/PM5/PP1/PP5 Likely pathogenic
LDLR	c.313+1G>A	Splice site	NA	2	PVS1/PM2/PM4 Pathogenic
LDLR	c.344G>A	Missense	p.Arg115His	3	PS1/PM2/PP3 Likely pathogenic
LDLR	c.361T>G	Missense	p.Cys121Gly	5	PM1/PM2/PP3/PP4/PP5 Likely pathogenic
LDLR	c.378del	Frameshift	p.Phe126LeufsTer80	4	PVS1/PM2/PM4/PP1 Pathogenic
LDLR	c.389dupC	Frameshift	p.Asp131ArgfsTer49	5	PVS1/PM1/PM2/PM4/PP5 Pathogenic

LDLR	c.413C>G	Nonsense	p.Ser138Ter	6	PVS1/PM2/PM4/PP1/PP5 Pathogenic
LDLR	c.418G>A	Missense	p.Glu140Lys	4	PM1/PM2/PP3/PP5 Likely pathogenic
LDLR	c.478T>C	Missense	p.Cys160Arg	4	PM1/PM2/PP3/PP5 Likely pathogenic
LDLR	c.489G>T	Missense	p.Gln163His	4	PM1/PM2/PP3/PP5 Likely pathogenic
LDLR	c.530C>T	Missense	p.Ser177Leu	4	PM1/PM2/PP3/PP5 Likely pathogenic
LDLR	c.532G>T	Missense	p.Asp178Tyr	5	PM1/PM2/PP3/PP5 Likely pathogenic
LDLR	c.611G>C	Missense	p.Cys204Ser	6	PM1/PM2/PP3/PP5 Likely pathogenic
LDLR	c.642G>C	Missense	p.Trp214Cys	7	PM1/PM2/PP3/PP5 Likely pathogenic
LDLR	c.682G>A	Missense	p.Glu228Gln	5	PM1/PM2/PP3/PP5 Likely pathogenic
LDLR	c.686_689del	Frameshift	p.Glu229AlafsTer35	4	PVS1/PM2/PM4/PP1 Pathogenic
LDLR	c.694+1G>A	Splice site	NA	2	PVS1/PM2/PM4/PP1 Pathogenic
LDLR	c.718G>A	Missense	p.Glu240Lys	3	PM1/PM2/PP3/PP5 Likely pathogenic
LDLR	c.718G>T	Nonsense	p.Glu240Ter	2	PM1/PM2/PP3/PP4 Likely pathogenic
LDLR	c.726G>C	Missense	p.Gln242His	4	PM1/PM2/PP3/PP4 Likely pathogenic
LDLR	c.796G>A	Missense	p.Asp266Asn	4	PM1/PM2/PP3/PP4 Likely pathogenic
LDLR	c.797A>G	Missense	p.Asp266Gly	3	PM1/PM2/PM5/PP1/PP3 Likely pathogenic
LDLR	c.829G>T	Nonsense	p.Glu277Ter	2	PVS1/PM2/PM4/PP1 Pathogenic

LDLR	c.874delC	Frameshift	p.Leu292TrpfsTer78	3	PVS1/PM2/PM4/PP1 Pathogenic
LDLR	c.901G>T	Missense	p.Asp301Tyr	5	PM1/PM2/PP3/PP5 Likely pathogenic
LDLR	c.902A>T	Missense	p.Asp301Val	4	PM1/PM2/PP3/PP5 Likely pathogenic
LDLR	c.937T>G	Missense	p.Cys313Gly	3	PM1/PM2/PP3/PP4 Likely pathogenic
LDLR	c.939C>A	Missense	p.Cys313Ter	2	PVS1/PM2/PM4/PP5 Pathogenic
LDLR	c.940+2T>C	Splice site	NA	2	PVS1/PM2/PM4/PP1 Pathogenic
LDLR	c.967G>A	Missense	p.Gly323Ser	4	PM1/PM2/PP3/PP4 Likely pathogenic
LDLR	c.1007_1010delACGA	Frameshift	p.Tyr336CysfsTer33	2	PVS1/PM2/PM4/PP5 Pathogenic
LDLR	c.1012T>A	Missense	p.Cys338Ser	2	PM1/PM2/PP3/PP5 Likely pathogenic
LDLR	c.1056C>A	Nonsense	p.Cys352Ter	4	PVS1/PM2/PM4/PP5 Pathogenic
LDLR	c.1062dupT	Frameshift	p.Ile355TyrfsTer3	5	PVS1/PM2/PM4/PP5 Pathogenic
LDLR	c.1067A>T	Missense	p.Asp356Val	4	PM1/PM2/PP3/PP4 Likely pathogenic
LDLR	c.1069G>T	Nonsense	p.Glu357Ter	2	PVS1/PM2/PM4 Pathogenic
LDLR	c.1114_1115insC	Frameshift	p.Glu372AlafsTer9	4	PVS1/PM2/PM4/PP5 Pathogenic
LDLR	c.1123_1124insGA	Nonsense	p.Tyr375Ter	2	PVS1/PM2/PM4/PP5 Pathogenic
LDLR	c.1183_1184insC	Frameshift	p.Val395AlafsTer46	2	PVS1/PM2/PM4/PP5 Pathogenic
LDLR	c.1187-2A>G	Splice site	NA	3	PVS1/PM2/PM4/PP5 Pathogenic

LDLR	c.1207T>C	Missense	p.Phe403Leu	2	PM1/PM2/PP3/PP5 Likely pathogenic
LDLR	c.1252G>A	Missense	p.Glu418Lys	6	PM1/PM2/PP3/PP4 Likely pathogenic
LDLR	c.1285G>A	Missense	p.Val429Leu	3	PM1/PM2/PP3/PP4 Likely pathogenic
LDLR	c.1297G>C	Missense	p.Asp433His	3	PM1/PM2/PP3/PP5 Likely pathogenic
LDLR	c.1328G>C	Missense	p.Trp443Ser	4	PM1/PM2/PP3/PP4 Likely pathogenic
LDLR	c.1339T>C	Missense	p.Ser447Pro	4	PM1/PM2/PP3/PP5 Likely pathogenic
LDLR	c.1340C>G	Missense	p.Ser447Cys	2	PM1/PM2/PP3/PP5 Likely pathogenic
LDLR	c.1432G>A	Missense	p.Gly478Arg	2	PM1/PM2/PP3/PP4 Likely pathogenic
LDLR	c.1466A>G	Missense	p.Tyr489Cys	2	PM1/PM2/PP3/PP5 Likely pathogenic
LDLR	c.1469G>A	Nonsense	p.Trp490Ter	1	PVS1/PM2/PM4/PP4 Pathogenic
LDLR	c.1474G>A	Missense	p.Asp492Asn	4	PM1/PM2/PP3/PP4 Likely pathogenic
LDLR	c.1567G>A	Missense	p.Val523Met	3	PM1/PM2/PP3/PP5 Likely pathogenic
LDLR	c.1573G>T	Missense	p.Asp525Tyr	2	PM1/PM2/PP3/PP4 Likely pathogenic
LDLR	c.1586+1G>A	Splice site	NA	2	PVS1/PM2/PM4/PP4 Pathogenic
LDLR	c.1652_1662delACATCTACTCG	Frameshift	p.Asp551AlafsTer4	4	PVS1/PM2/PM4/PP4 Pathogenic
LDLR	c.1702C>G	Missense	p.Leu568Val	4	PM1/PM2/PP3/PP5 Likely pathogenic
LDLR	c.1705+1G>C	Splice site	NA	4	PVS1/PM2/PM4/PP4 Pathogenic

LDLR	c.1706A>G	Missense	p.Asp569Gly	2	PM1/PM2/PP3/PP4 Likely pathogenic
LDLR	c.1727A>G	Missense	p.Tyr576Cys	6	PM1/PM2/PP3/PP4 Likely pathogenic
LDLR	c.1731G>T	Missense	p.Trp577Cys	6	PM1/PM2/PP3/PP4 Likely pathogenic
LDLR	c.1778dupG	Frameshift	p.Asn594GlnfsTer9	5	PVS1/PM2/PM4/PP4 Pathogenic
LDLR	c.1783C>T	Missense	p.Arg595Trp	4	PM1/PM2/PP3/PP4 Likely pathogenic
LDLR	c.1845+2T>C	Splice site	NA	43	PVS1/PM2/PM4/PP4 Pathogenic
LDLR	c.1859G>C	Missense	p.Trp620Ser	2	PM1/PM2/PP3/PP4 Likely pathogenic
LDLR	c.1868T>A	Missense	p.Ile623Asn	3	PM1/PM2/PP3/PP4 Likely pathogenic
LDLR	c.1897C>T	Missense	p.Arg633Cys	2	PM1/PM2/PP3/PP4 Likely pathogenic
LDLR	c.1925T>C	Missense	p.Leu642Ser	3	PM1/PM2/PP3/PP4/PP5 Likely pathogenic
LDLR	c.1998G>C	Missense	p.Trp666Cys	2	PM1/PM2/PP3/PP4 Likely pathogenic
LDLR	c.2050G>T	Missense	p.Ala684Ser	2	PM1/PM2/PP3/PP4 Likely pathogenic
LDLR	c.2054C>T	Missense	p.Pro685Leu	34	PM1/PM2/PP3/PP4/PP5 Likely pathogenic
LDLR	c.2096C>T	Missense	p.Pro699Leu	4	PM1/PM2/PP3/PP4 Likely pathogenic
LDLR	c.2389G>A	Missense	p.Val797Met	13	PM1/PM2/PP3/PP4 Likely pathogenic
LDLR	c.2390-4_2393delACAGTGCT	Splice site	NA	3	PVS1/PM2/PM4/PP4 Pathogenic
LDLR	c.2416delG	Frameshift	p.Val806SerfsTer123	4	PVS1/PM2/PM4/PP4 Pathogenic

LDLR	c.2416dupG	Frameshift	p.Val806GlyfsTer11	4	PVS1/PM2/PM4/PP4 Pathogenic
LDLR	c.2431A>T	Nonsense	p.Lys811Ter	185	PVS1/PM2/PM4/PP4 Pathogenic
LDLR	c.2500G>A	Missense	p.Asp834Asn	4	PVS1/PM2/PM4/PP4 Pathogenic
LDLR	c.2579C>T	Missense	p.Ala860Val	4	PVS1/PM2/PM4/PP4 Pathogenic
LDLR	c.313-?_2311+?del	Large deletion	Truncated protein	21	PVS1/PM2/PM4/PP4 Pathogenic
LDLR	c.1186-?_1587+?dup	Large duplication	Truncated protein	17	PVS1/PM2/PM4/PP4 Pathogenic
LDLR	c.1845-?_2141+?del	Large deletion	Truncated protein	16	PVS1/PM2/PM4/PP4 Pathogenic
LDLR	c.2141-?_2311+?del	Large deletion	Truncated protein	15	PVS1/PM2/PM4/PP4 Pathogenic
PCSK9	c.94G>A	Missense	p.Glu32Lys	42	PS1/PS3/PP3/PP4/PP5 Pathogenic

ACMG: American College of Medical Genetics, *LDLR*: low-density lipoprotein receptor, *PCSK9*: proprotein convertase subtilisin/kexin type 9, NA: not applicable, FH: familial hypercholesterolemia

Supplemental Table 2. Characteristics of the study subjects included and excluded

Variable	Study population (n = 1,050)	Individuals excluded* (n = 955)	p value
Age (years)	49 ± 16	44 ± 19	0.0034
Male gender (%)	490 (46.7%)	448 (46.9%)	0.95
LDL cholesterol level (at baseline, mg/dL)	244 [208–279]	240 [200–268]	0.14

*homozygous and compound heterozygous FH were excluded.
